# Application of platelet-rich plasma in rectovaginal fistulas in the patients with ulcerative colitis

**DOI:** 10.1007/s00423-021-02232-7

**Published:** 2021-06-21

**Authors:** Jacek Hermann, Jarosław Cwaliński, Tomasz Banasiewicz

**Affiliations:** Department of General, and Endocrynologic Surgery, and Gastroenterologic Oncology, Poznań University of Medical Sciences, 49 Przybyszewski Str., 60-355 Poznań, Poland

**Keywords:** Platelet-rich plasma, Rectovaginal fistulas, Inflammatory bowel diseases

## Abstract

**Background:**

There is a need for other than surgical methods of therapy for small and low rectovaginal fistulas (RVF) in the course of inflammatory bowel diseases (IBD), such as application of fibrin sealants, stem cells, biological therapy, or platelet-rich plasma. The aim of this study was to evaluate the results of the treatment after local application of PRP in aforementioned fistulas, exclusively in the patients with ulcerative colitis (UC).

**Patients and methods:**

Medical records of 13 patients with small and low-lying, active RVF in the course of UC, and after restorative proctocolectomy for UC were evaluated. Curettage of fistulous tracts was performed with the following application of PRP in all patients.

**Results:**

Complete closure of RVF was achieved after the first injection in 4 patients, 3 women healed their fistulas following the second application, and two of them closed RVF after 3 injections. To sum up, the complete closure of RVF was achieved in 9 (69%) patients. Fistulas remained closed from 6 to 12 months.

**Conclusions:**

The application of PRP in small, low, and recurrent IBD anal fistulas is effective, simple, and safe with an acceptable rate of healing. This therapy might also precede any further, surgical methods of treatment.

## Introduction

Rectovaginal fistula (RVF) is referred to as an epithelium-lined abnormal communication between the rectum and vagina. Although uncommon, it represents a considerable burden on the physical and mental health of a woman, as well as a major challenge for a surgeon. Although the overwhelming majority of those fistulas are caused by obstetric injuries, inflammatory bowel diseases (IBD) may also result in fistula formation. RVF affect more commonly the patients with Crohn’s disease (CD), compared to those with ulcerative colitis (UC) [1]. The fistula may develop either in the course of IBD, or after restorative proctocolectomy (IPAA), performed more frequently for UC. Differentiation between the Crohn’s and ulcerative colitis fistula is difficult in some cases, as well as between the IBD fistula and a cryptoglandular one. Perhaps, the fistula develops in the course of UC from a deep ulcer penetrating the rectovaginal septum with following abscess, and then with the fistula formation. Initial diagnosis of the UC fistula may change in time into the Crohn’s one in 30% of the patients as a result of evolving natural history of the disease. The iatrogenic fistula is a consequence of damage to the vaginal wall which appears during either resection of the rectum or after false anastomosis between the ileal “J-pouch” with remnant of the rectum, and with the vagina interposition between both gastrointestinal remnants and a false anastomosis of the pouch with the vagina [2, 3]. Based on the size of a defect within the rectovaginal septum, small, medium, or large fistulas are distinguished. The diameter of the small one is estimated below 5 mm. Location of the fistula’s opening in both the rectum or the vagina determines the further classification of fistulas into either low or high. The tract of a low fistula runs within or below the anal sphincter, whereas the rectal opening of the fistula is located either above or below the pectinate line. The latter are classified also as ano-vaginal fistulas. The vaginal orifice of a low RVF opens just inside the vaginal fourchette or in the lower part of the vagina. Clinical presentation of RVF depends on the size and localization of orifices, and stool consistency. It is manifested by the passage of gases, feces, or purulent discharge out of the vagina and by recurrent infections of the vagina or urinary tract. In most cases, a large fistula can be diagnosed with digital rectal examination or the rectal and vaginal examination. The rectovaginal septum defect is usually palpable or presents visually either in the form of an orifice that connects the vagina with rectum, anus, or in the form of a dimple in the rectal or vaginal mucosa. Small fistulas can be confirmed by dyeing of a tampon inserted into the vagina following methylene blue enema, or by contrast studies. Physical examination should be followed by endoscopy with possible biopsy specimen of the mucosa in the orifice vicinity [4]. The patients with IBD anal fistulas developing in the course of the disease are treated in conservative manner to achieve at least palliation of their symptoms such as alleviation of pain and reduction of discharge from the fistula orifice. Complete closure of an anal fistula is the definitive objective of the treatment. Several sphincter-preserving procedures have been used, e.g., mucosal advancement flaps (MAF), or video-assisted anal fistula treatment (VAAFT). The rate of RVF healing ranged from 50 to 60% after the former procedure, compared to over 70% after the latter. However, the promising early results after VAAFT require further evidence [5, 6]. It is worth emphasizing that the abovementioned surgical procedures should be combined with the biological therapy. It is estimated that the rate of complete fistula closure reaches 50% even with the biological therapy alone [7]. The patients with iatrogenic RVF fistulas require either local repair with trans-anal or trans-vaginal approach preceded with loop ileo- or colostomy, or they require ileal-pouch-rectal reanastomosis after the dismantlement of the previous anastomosis. In case of recurrent fistulas that tend to damage the surrounding tissues considerably, the rectovaginal septum can be reconstructed with the use of muscles, bowel, or mesh implant [8]. However, there are also alternative, conservative methods of treatment indicated for recurrent, low, and small fistulas with attenuated, scarred septum with poor blood supply, e.g., application of fibrin sealants, stem cells, or platelet-rich plasma (PRP) [9]. The aim of this study was to evaluate the results of the treatment after local application of PRP in the aforementioned fistulas, exclusively in the patients with UC.

## Patient and methods

**Fig. 1:**
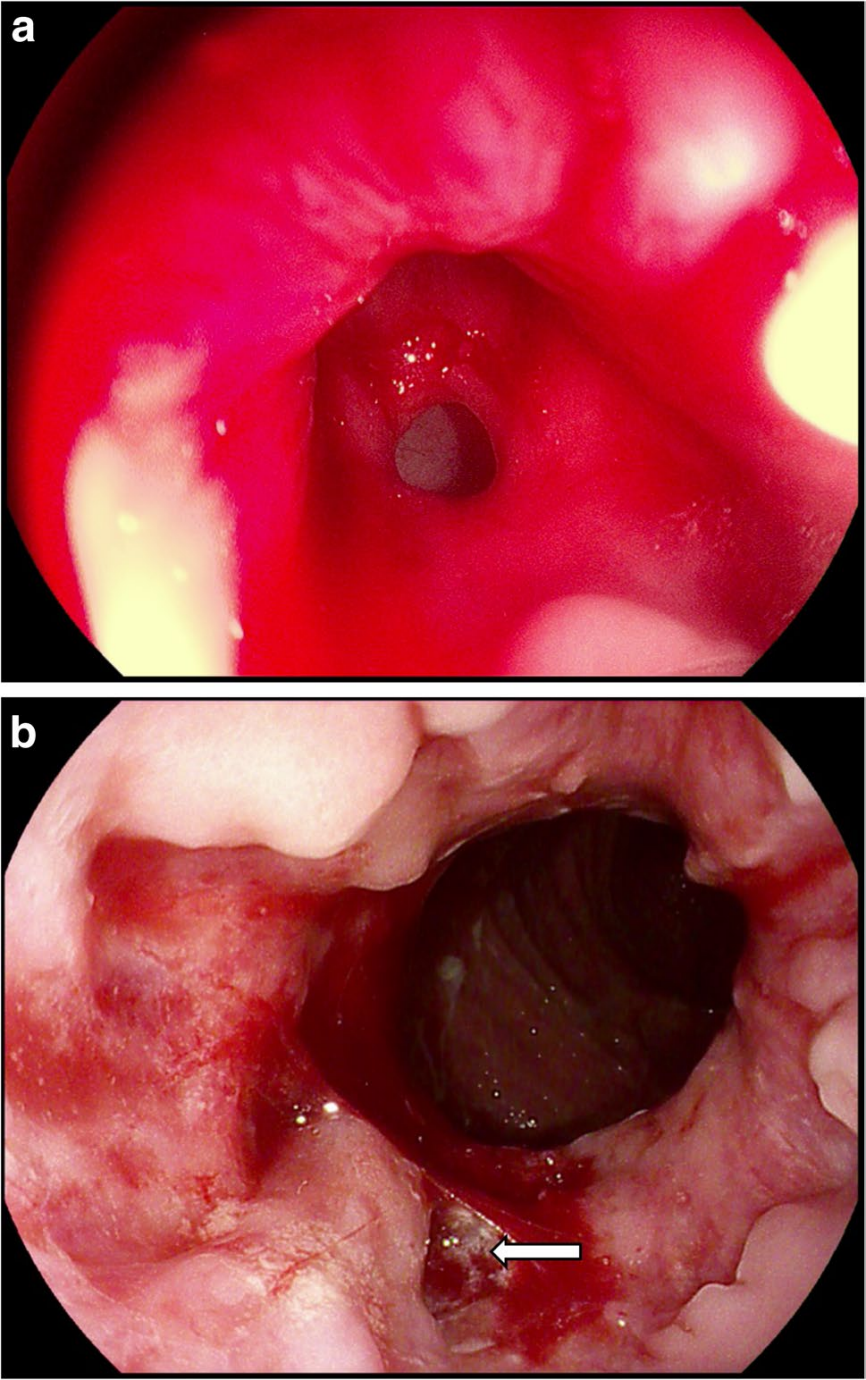
Endoscopic evaluation of rectovaginal fistula. **A** Vaginal orifice. **B** Rectal orifice with bleeding

**Fig. 2:**
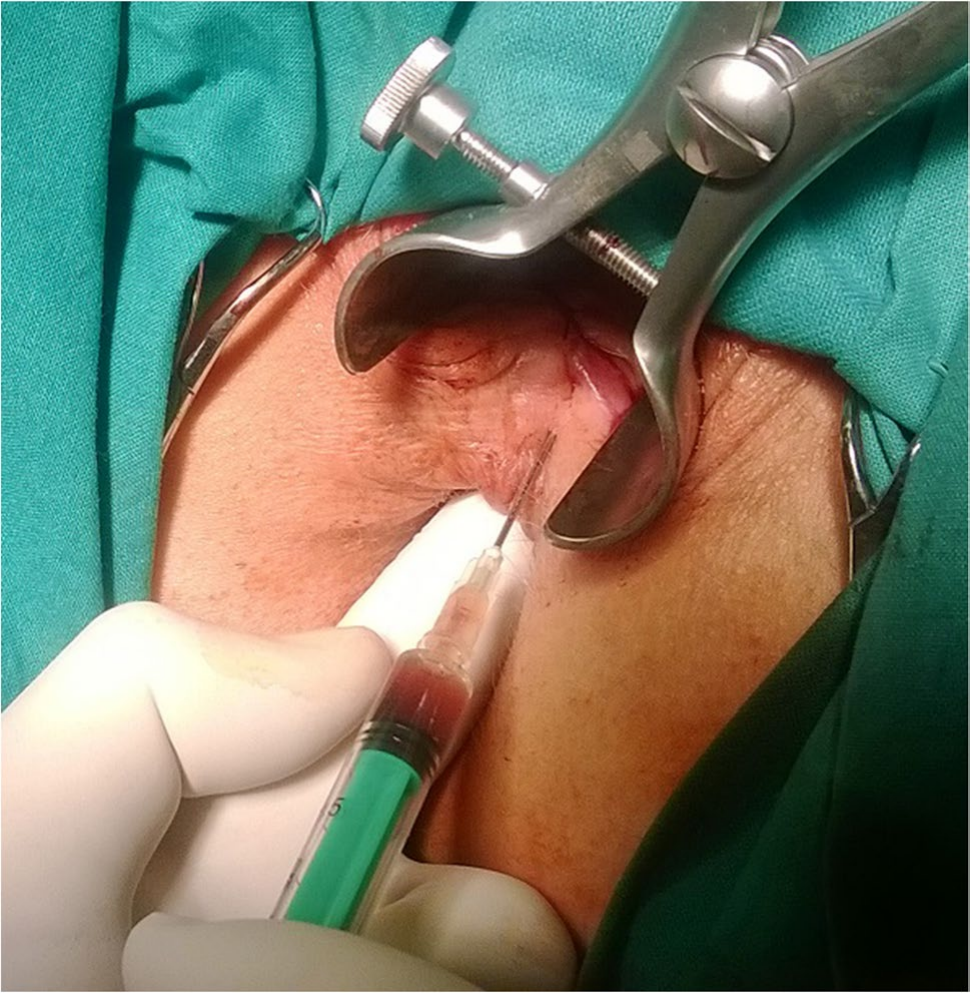
Penetration depth controlled with index inserted into vagina and anal retractor

**Fig. 3:**
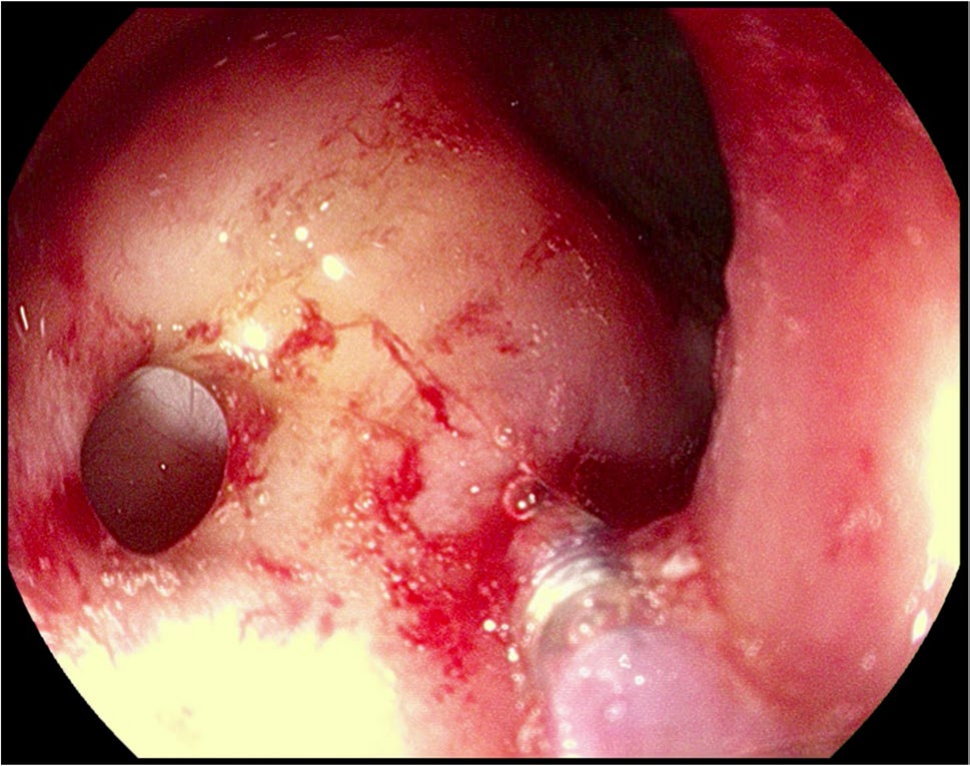
Application of PRP with endoscopic measures in a higher fistula

Medical records of 13 patients with small and low-lying, active RVF in the course of UC, and following restorative proctocolectomy for UC, treated for RVF, between 2018 and 2020, were evaluated. Patients with RVF due to other causes such as Crohn’s disease, obstetric injuries, crypto-glandular infections, radiation, gynecologic malignancies, and surgery were excluded from this study (Table 1). The mean age was 44 (IQR 36–54 years). Seven of them underwent IPAA before, and the fistulas failed to close with local repair in 7 patients, and in 6 women after MAF. The group comprised 6 women with ileostomy (Table 2). The patients underwent digital rectal and vaginal examination, as well as probing and/or dyeing of fistulous tracts during endoscopic visualization, under oral premedication or general anesthesia (Fig. 1A, B). Curettage of fistulous tracts to remove the epithelium and infected granulation tissue with following irrigation of those canals was performed in all patients. This procedure was accomplished with the application of PRP. The volume of 15 ml of blood was centrifuged with the speed of 1776* g* to obtain 1 ml of PRP with concentration of 10^6^ of platelets, using a commercial kit (Xerthra PRP Kit, Biovico, Poland). The plasma was injected into the tissues surrounding fistulous tract, beginning from the anal orifice towards the vaginal one. The penetration depth was controlled with the index inserted into the vagina or with TRUS (Fig. 2). In higher fistulas, PRP was applied with endoscopic measures (Fig. 3). The procedure was repeated 3 times every fortnight in accordance with the protocol until closure of fistula was achieved with the possibility to double the number of applications if it was clinically justified.

**Table 1:** Etiology of rectovaginal fistulas

Etiology	Number (%)	
Crohn’s disease		15 (34)
Obstetric		7 (16)
IPAA for UC		7 (16)
Ulcerative colitis		6 (14)
LAR		4 (9)
Cryptoglandular infection		2 (4)
Radiation		2 (4)
Hysterectomy		1 (2)
Total		44

*IPAA* ileal pouch anal anastomosis,* UC* ulcerative colitis,* LAR* low anterior resection

**Table 2:** Characteristics of patients

Patients		N = 13	Percent
Age (in years)		Me = 44 (range 36–54)	
Previous surgery		13	100
	IPAA	7	54
	Ileostomy	6	46
	Local repair	7	54
	MAF	6	46
Medical treatment		13	100
	Antibiotics	13	100
	Infliximab	5	38
	Adalimumab	2	15

*MAF* mucosal advancement flap

## Results

**Fig. 4:**
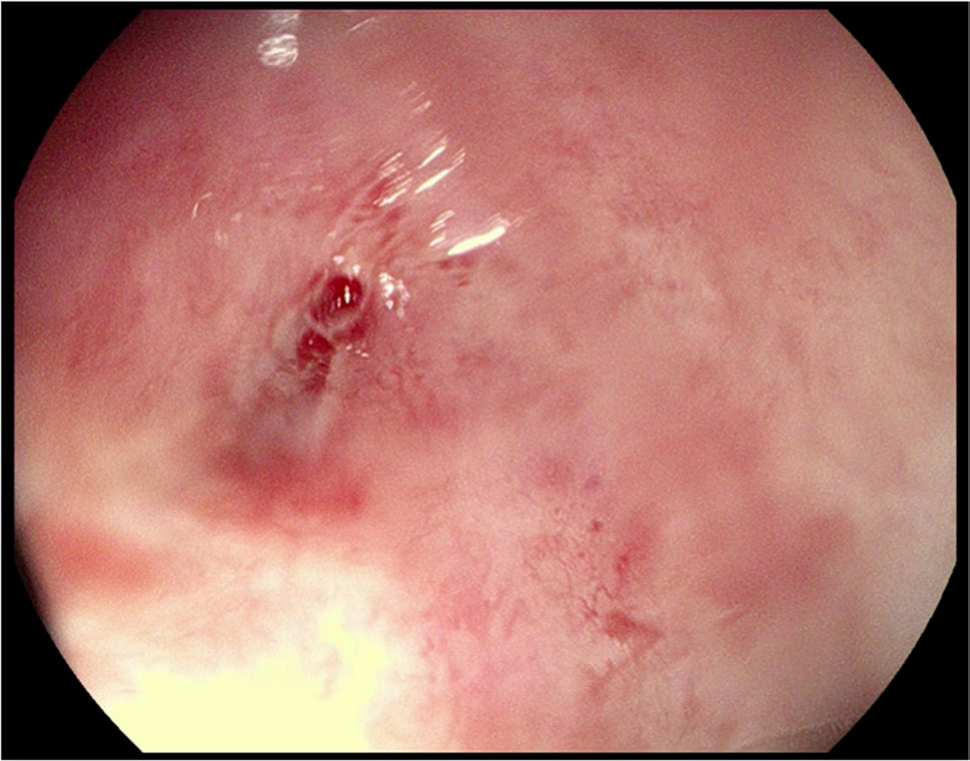
Rectovaginal fistula healed in 12 months after therapy

The investigated patients were evaluated in an out-patient department after fortnight and then in 1, 6, and 12 months following the final PRP application (Fig. 4). That follow-up lasted from 6 to 23 months, whereas the median follow-up was 12 months long. That evaluation consisted of digital rectal examination, and TRUS. Complete closure of RVF was achieved in 4 patients after the first injection, 3 women healed their fistulas following the second application, and two of them closed RVF after 3 injections. To sum up, the complete closure of RVF was achieved in 9 (69%) women. Fistulas remained closed from 6 to 23 months. There were no other complications in the surveyed group of patients.

## Discussion

Application of fibrin sealants, stem cells, and platelet-rich plasma (PRP) for small rectovaginal fistulas (RVF) seem to be simple, painless, and with low rate of morbidity. However, it is difficult to assess definitive effectiveness of those methods due to the lack of randomized, controlled trials as well as case reports, and case series. Therefore, the results of treatment with, e.g., PRP can be compared to application of the plasma for similar indications or to other conservative methods of treatment. PRP has been used today more often as supportive therapy to reinforce suture lines following advancement flap for anal fistulas or PRP was injected into the tissues surrounding vesico-vaginal fistulas to restore the tissue’s ability to heal prior to surgery. It is also applied for skin grafts as an effective adhesive. In other words, the plasma creates a matrix to which the graft can bind [10]. However, it is possible to compare the results of PRP application in RVF to the use of the plasma in anal fistulas since several case reports and case series regarding that indication have been already obtained. PRP is an autologous product obtained from the whole blood through the process of gradient density centrifugation. The results achieved after application of PRP, in the investigated group of patients, were better compared to other discussed reports with complete closure of RVF in almost 70% of patients. The higher efficacy, compared to other conservative methods, is probably due to the use of three ways of PRP action. On the one hand, the plasma works as a fibrin tissue adhesive, and a scaffold for cellular infiltration. On the other hand, it promotes wound healing by increasing cellular proliferation, angiogenesis, and collagen synthesis because of a high concentration of platelet-derived growth factors within the soft tissues in vicinity of fistulous tracts. Mechanical compression of the fistulous tract by the plasma, injected into the adjacent tissues to anal fistulas, is an additional PRP mechanism of action [6]. As far as the number of PRP injections is considered, fistulas were clinically closed following 2 to 3 injections. The average time of recurrence was reported at 6–9 weeks after final application of the plasma. Application of PRP in Crohn’s anal fistulas was reported by de la Portilla to be safe, with the healing rate below 50%. The rate increased, if the biological therapy was used concomitantly. The relatively low rate of fistula closure could have been a result of administration of the plasma exclusively into the fistulous canal [11]. Complete healing of anal fistulas after PRP application reached 62% in a study reported by Moreno-Serrano. Although the plasma was injected directly into the fistulous canal, internal orifice was additionally closed with a couple of sutures [12]. As far as fibrin glue is considered, a separately packed thrombin is mixed with fibrinogen to form a fibrin clot, which seals a fistulous tract. That clot promotes fistula healing after gradual fibrinolysis. That bio-product is introduced exclusively into a fistulous tract, beginning from the anal orifice after curettage of the canal. A major advantage of this method is the opportunity to repeat it if a failure occurs, or possible implementation of other methods of surgery in the future, if necessary [13]. Low efficacy of the method resulting from a frequent expulsion of the clot, estimated at approximately 50%, is a major drawback of this approach. However, it is worth mentioning that reports concerning RVF therapy with fibrin sealants have not yet been made available [14]. The high rate of the fibrin clot displacement, in a few days after application, is probably a consequence of introducing the glue directly into the fistulous canal, and a generally short length of the tracts within RVF. This hypothesis was put forward by Sentovich [15]. Mesenchymal stem cells obtained from human adipose tissue provide a way to increase the number of locally deficient cells, and to restore normal wound healing. Those cells are used also due to their immunomodulatory mode of action. Stem cells are administered into the tissues surrounding fistulous tracts. Preliminary results of this treatment regarding RVF are encouraging. However, further properly designed studies are necessary [16].

## Conclusions

The application of PRP in small, low, and recurrent IBD anal fistulas is effective, simple, and safe with an acceptable rate of healing. This therapy might also precede any further, surgical methods of treatment.
